# Retroperitoneal cystic mass: a rare form of adrenal pheochromocytoma

**DOI:** 10.1093/jscr/rjab169

**Published:** 2021-05-06

**Authors:** Rahoui Moez, Yassine Ouanes, Wajih Sahnoun, Bibi Mokhtar, Khaireddine Mrad Dali, Ahmed Sellami, Sami Ben Rhouma, Yassine Nouira

**Affiliations:** Departement of Urology, La Rabta Hospital, Tunis, Tunisia; Departement of Urology, La Rabta Hospital, Tunis, Tunisia; Departement of Urology, La Rabta Hospital, Tunis, Tunisia; Departement of Urology, La Rabta Hospital, Tunis, Tunisia; Departement of Urology, La Rabta Hospital, Tunis, Tunisia; Departement of Urology, La Rabta Hospital, Tunis, Tunisia; Departement of Urology, La Rabta Hospital, Tunis, Tunisia; Departement of Urology, La Rabta Hospital, Tunis, Tunisia

## Abstract

Adrenal cysts are usually non-functional and asymptomatic. Cystic pheochromocytomas are a rare clinical entity and difficult to differentiate from simple cysts in the absence of classic clinical symptoms. Few cases of cystic pheochromocytomas have been reported in the literature. We present a case of a huge cystic pheochromocytoma in 70-year-old men who presented with a large retroperitoneal cystic mass and discuss difficulties of diagnostic and treatment.

## INTRODUCTION

Pheochromocytomas are tumors of pronounced clinical importance. Its incidence is 0.1% in the general population [2]. Usually, it is a solid neoplasm of the adrenal medulla; however cystic pheochromocytoma is a rare neuroendocrine tumor that is frequently asymptomatic. Few cases of cystic pheochromocytomas have been reported in the literature [1]. Considering the rarity and difficulty to differentiate from simple cysts in the absence of classic clinical symptoms, we present a huge cystic pheochromocytoma and discuss the diagnostic and treatment.

## CASE REPORT

**Figure 1:**
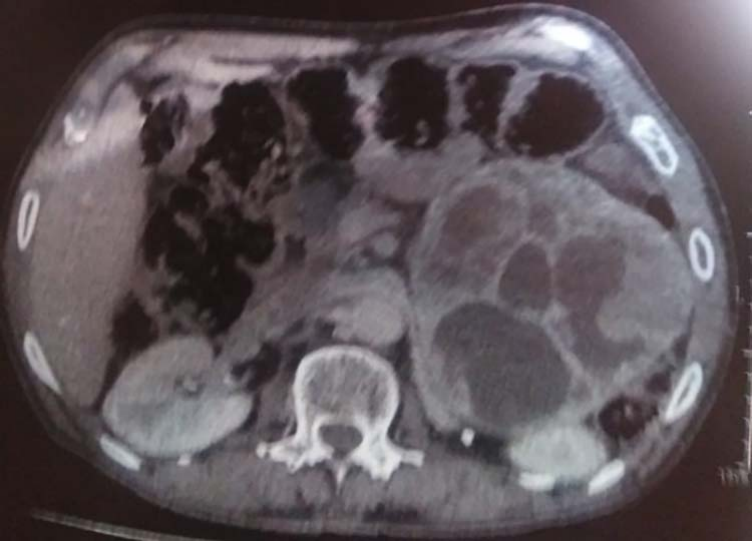
CT scan image showing left retroperitoneal cyst.

**Figure 2:**
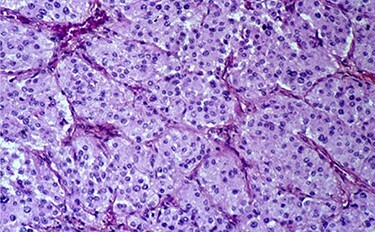
Histopathological section showing round to polygonal cells forming a zellballen.

**Figure 3:**
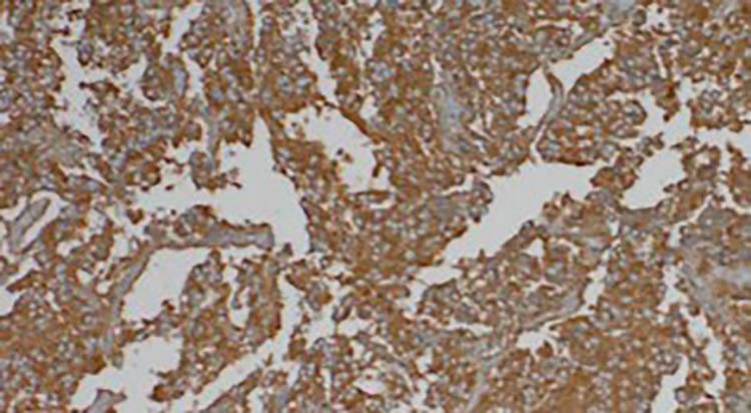
Immunohistochemically staining showing cells positive for chromogranin A.

A 70-year-old patient, diabetic on insulin who presented with left flank pain evolving for 6 months. Medical history showed only the presence of headaches. The clinical examination showed a mass of the left flank, a pulse at 95 bpm and elevated blood pressure of 185/95 mmHg. Computed tomography (CT) showed a left retroperitoneal cystic mass measuring 13*11 cm on the axial plane with a large axis of 13 cm with a fleshy component and moderate enhancement. The left adrenal gland was not found (Fig. 2). Laboratory investigations were normal. A screening hormone test related to the adrenal gland, revealed elevated plasma catecholamine levels, epinephrine, 0.61 ng/mL (*N*: 0.00–0.10); norepinephrine, 2.88 ng/ml (*N*: 0.10–0.50). Furthermore, detailed 24-h urinalyses during hospitalization showed elevated urinary catecholamine levels, norepinephrine, 995.4 mg/24 h (N: 31.0–160.0), metanephrine levels with metanephrine, 2.35 mg/24 h (N: 0.04–0.18); normetanephrine, 3.75 mg/24 h (N: 0.10–0.28; Table 1). Based on these findings, the diagnosis of cystic pheochromocytoma was suspected. The patient’s blood pressure was controlled with the alpha-blocker. The patient was taken up for exploratory laparotomy. Intraoperatively, a giant cystic mass was found arising from the left adrenal gland and was adherent to the left renal pedicle and pancreas. Manipulation of the mass resulted in extreme blood pressure fluctuations. The patient underwent removal of this adrenal cystic tumor. Macroscopically, the mass measures 15 × 12 × 12 cm, round, well-circumscribed and encapsulated. It had a central cystic space filled with hemorrhagic fluid. Histologically, the tumor was confirmed as a pheochromocytoma, and no malignant foci were detected (Fig. 3). Immunohistochemical staining revealed that the tumor cells were diffusely positive for chromogranin A (Fig. 1). The patient had an uneventful recovery and was discharged on the fifth postoperative day. Three months following surgery, the patient remains stable with no tumor recurrence and his most recent ambulatory blood pressure was 114/67 mmHg with low-dose amlodipine. After 6 months of follow-up, his 24-h urine metanephrine and normetanephrine were within a normal range.

**Table 1:** Laboratory parameters

Variables	Values	Normal values
Epinephrine	0.61 ng/ml	0.00–0.10
Norepinephrine	2.88 ng/ml	0.10–0.50
24-h urine metanephrine	2.35 mg/24 h	0.04–0.18
24-h urine normetanephrine	3.75 mg/24 h	0.10–0.28
24-h urine norepinephrine	995.4 mg/24 h	31.0–160.0

## DISCUSSION

Pheochromocytoma is a rare neuroendocrine tumor. It was a tumor arising from adrenomedullary chromaffin cells that commonly produce one or more catecholamines. They produce and secrete catecholamine; the triad of headache, sweating and palpitations in patients with hypertension is diagnostic, with a 94% specificity and 91% sensitivity [3]. The frequency reaches 1/10 000 cases in the general population [2]. A typical pheochromocytoma presents as a solid lesion that can be easily diagnosed with available laboratory analyses and imaging investigations, but it is difficult to diagnose a cystic pheochromocytoma because it structurally resembles a benign adrenal cyst. It can cause multiple signs and symptoms such as headache, palpitations and hypertension but some patients with pheochromocytomas may be normotensive and asymptomatic [4]. Cystic pheochromocytoma is a rare entity and it may be due to intralesional hemorrhage, necrosis and liquefaction. Patients with cystic pheochromocytomas are usually asymptomatic. Hormonal analysis is generally negative [5]. Our patient presented headache and hypertensive crisis and the hormonal analysis objectified an elevation of the blood and urinary methoxylated derivatives. The radiological study of the adrenal gland is based on CT. The typical radiological characteristics of pheochromocytoma are a round or oval mass, well-circumscribed, homogeneous or heterogeneous measuring > 4 cm, increased attenuation on unimproved enhancement, significant vascularization of the mass [1, 6]. However, the CT characteristics of cystic pheochromocytomas would be a relatively thick wall, the presence or absence of septa in the mass and persistent enhancement of the wall after the administration of contrast media [4]. On the other hand, 123I-MIBG scintigraphy has a higher diagnostic specificity (95–100% for pheochromocytoma). 18F-FDG positron emission tomography/CT can also allow the successful visualization of pheochromocytomas [7]. Preoperative management is essential to prevent hemodynamic instability during the intraoperative or postoperative period. The treatment of cystic pheochromocytomas is based on surgery to avoid any potential hypertensive crisis and a variety of cardiovascular complications. Although some reports have concluded that laparoscopic adrenalectomy is safe, effective and minimally invasive, it is considered a gold standard in the surgical management of small benign adrenal tumors. Essential intraoperative surgical steps include early isolation of the tumor’s venous drainage with minimal manipulation of the mass followed by complete resection of the tumor. In immunohistochemistry, the tumor cells are reactive for, chromogranin A, synaptophysin and S-100 confirming the diagnosis of pheochromocytoma [4]. Patients should be followed-up yearly for at least 10 years, as 16% of patients develop recurrence within 10 year [5, 8].

## CONCLUSION

Cystic pheochromocytoma is a rare entity and mostly asymptomatic. The clinical course of cystic pheochromocytoma is similar to that of solid tumor, however cystic mass imposesimposes clinical and radilogical and horomnal investigations for the preoperative diagnosis of cystic pheochrmocytoma. Surgical resection is the only treatment for such tumors after blood pressure control with an alpha-blocker.

## References

[1] Sebastiano C , Zhao X, Deng FM, Das K. Cystic lesions of the adrenal gland: our experience over the last 20 years. Hum Pathol 2013 Sep;44:1797–803. 23618356 10.1016/j.humpath.2013.02.002

[2] Adler JT , Meyer-Rochow GY, Chen H, Benn DE, Robinson BG, Sippel RS, et al. Pheochromocytoma: current approaches and future directions. Oncologist 2008;13:779–93. 18617683 10.1634/theoncologist.2008-0043

[3] Bush WH , Elder JS, Crane RE, Wales LR. Cystic pheochromocytoma. Urology 1985;25:332–4. 3976127 10.1016/0090-4295(85)90346-2

[4] Andreoni C , Krebs RK, Bruna PC, Goldman SM, Kater CE, Alves MT, et al. Cystic phaeochromocytoma is a distinctive subgroup with special clinical, imaging and histological features that might mislead the diagnosis. BJU Int 2008;101:345–50. 18070168 10.1111/j.1464-410X.2007.07370.x

[5] Mohammed S , Vijaya G, Priyanka S, Raghunath K, Janakir A, Shivshankar S. Cystic Pheochromocytoma presenting as adrenal cyst. J Clin Diagn Res 2016;10:OD09–10. 10.7860/JCDR/2016/20129.8892 PMC5198380 28050427

[6] Munden R , Adams DB, Curry NS. Cystic pheochromocytoma: radiologic diagnosis. South Med J 1993;86:1302–5. 8235793 10.1097/00007611-199311000-00029

[7] Pacak K , Linehan WM, Eisenhofer G, Walther MM, Goldstein DS. Recent advances in genetics, diagnosis, localization, and treatment of pheochromocytoma. Ann Intern Med 2001;134:315–29. 11182843 10.7326/0003-4819-134-4-200102200-00016

[8] Basiri A , Radfar MH. Giant cystic pheochromocytoma. Urol J 2010;7:16. 20209449

